# Foetal lipoprotein oxidation and preeclampsia

**DOI:** 10.1186/s12944-022-01663-5

**Published:** 2022-06-04

**Authors:** LA Gil-Acevedo, Guillermo Ceballos, YD Torres-Ramos

**Affiliations:** 1 Laboratorio Central, Instituto Nacional de Perinatología Isidro Espinosa de los Reyes, Secretaría de Salud, Montes Urales 800, Lomas Virreyes, Miguel Hidalgo, 11000 Ciudad de México, Mexico; 2 Escuela Superior de Medicina, unidad de posgrado, Instituto Politécnico Nacional, Salvador Díaz Mirón, Esq. Plan de San Luis S/N, Miguel Hidalgo, Casco de Santo Tomas, 11340 Ciudad de México, México; 3 Laboratorio de Investigación Integral Cardiometabólica, Escuela Superior de Medicina, Instituto Politécnico Nacional, Salvador Díaz Mirón, Esq. Plan de San Luis S/N, Miguel Hidalgo, Casco de Santo Tomas, 11340 Ciudad de México, México; 4 Departamento de Inmunobioquímica, Instituto Nacional de Perinatología Isidro Espinosa de los Reyes, Secretaría de Salud. Montes Urales 800, Lomas Virreyes, Miguel Hidalgo, 11000 Ciudad de México, México

**Keywords:** Lipoproteins, Oxidative damage, Preeclampsia, Foetal programming

## Abstract

Preeclampsia (PE) is a multisystemic syndrome specific to pregnancy. Although PE is the leading cause of death from complications associated with pregnancy, its aetiology is still unknown. In PE, lipid metabolism is altered. When lipids are damaged, both the mother and the foetus may be at risk. Lipoproteins contain apolipoproteins, triacylglycerols, free and esterified cholesterol, and phospholipids, all of which are susceptible to oxidative stress when high levels of oxygen and nitrogen free radicals are present. Lipoperoxidation can occur in three stages: mild, moderate, and severe. In severe lipid damage, highly toxic products such as malondialdehyde (MDA) can be generated; under these conditions, low-density lipoprotein (LDL) proteins can be oxidized (oxLDL). oxLDL is a biomolecule that can affect the production of nitric oxide (NO), the main vasodilator derived from the endothelium. oxLDL can interfere with the transduction of the signals responsible for triggering the activation of endothelial nitric oxide synthase (eNOS), causing reduced vasodilation and endothelial dysfunction, which are the main characteristics of preeclampsia. The objective of the review was to analyse the information the current information about exists about the impact generated by the oxidation of LDL and HDL lipoproteins in neonates of women with preeclampsia and how these alterations can predispose the neonate to develop diseases in adulthood.

PE can cause foetal loss, intrauterine growth restriction, or developmental complications. Neonates of mothers with PE have a high risk of cardiovascular diseases, stroke, mental retardation, sensory deficiencies and an increased risk of developing metabolic diseases. PE not only affects the foetus, generating complications during pregnancy but also predisposes them to chronic diseases in adulthood.

## Introduction

Preeclampsia (PE) is a syndrome that occurs during pregnancy after the 20th week of gestation. It is classified according to its severity (Table 1), [1–3]; and subclassified according to the time of appearance of its signs and symptoms (Table 2), [4, 5].

**Table 1:** Classification of Preeclampsia

Classification	Diagnostic criteria	Reference
Preeclampsia	Blood pressure: systolic/diastolic ≥ to 140/90 mmHg. (2 shots 4 h apart) -Proteinuria: ≥ to 300 mg in 24-h urine -Thrombocytopenia (less than 100 cells/mm3) -Altered liver function By determining: []TGO (oxalacetic transaminase or aspartate aminotransferase, AST) []TGP (pyruvic transaminase or alanine aminotransferase, ALT) [Greater] 5 times the normal value (5–35 IU) = 180 IU -Alteration in kidney function Creatinine excretion > 1.2 mg/dL	[1–3]
Preeclampsia with severe features	Blood pressure: systolic/diastolic ≥ to 160/110 mm Hg -Proteinuria > 300 mg in a 24-h urine collection -Thrombocytopenia -Impaired renal function -Impaired liver function -Others: Pulmonary oedema, cerebral or visual symptoms	[1–3]

**Table 2:** Subclassification of Preeclampsia

Subclassification	Signs and symptoms	Reference
Early onset PE (eoPE)	Manifestations and delivery < 34 weeks of gestation	[4, 5]
Premature PE	Manifestations and delivery < 37 weeks of gestation	[4]
Late onset PE (loPE)	Manifestations and delivery ≥ 34 weeks of gestation	[4, 5]
Term PE	Manifestations and delivery ≥ 37 weeks of gestation	[4]

According to the classification of the International Federation of Gynecology and Obstetrics, preeclampsia is subclassified according to the manifestations and resolution of delivery; in early-onset PE (eoPE), they start before the 34th week of gestation; in premature PE, they start before the 37th week of gestation; in late-onset PE (loPE), they begin at or after the 34th week of gestation; and in term PE, they start at or after the 37th week of gestation. Marin et al. only subclassifies early-onset PE (eoPE) and late-onset PE (loPE).

Multiple studies have focused on analysing the role that lipoproteins play in PE and how the modification or damage that these molecules may endure affects neonates in adult life [6, 7]. In the 1980s, a foetal programming concept emerged that suggested that the intrauterine environment determines and programmes physiology and metabolism throughout life [8]. Lipids play an important role during pregnancy, and in this period complex changes occur in lipoprotein metabolism [9]. In the first months, anabolic metabolism prevails, increasing fat deposits. In this stage, the accumulation of fat is mediated essentially by maternal hyperphagia, which facilitates the exogenous supply of lipids with an increase in lipoprotein lipase (LPL) activity; in this way, the ingested lipids can be transferred to the foetus [10, 11].

However, in the third trimester of pregnancy, which is also when foetal growth is greatest, overall LPL activity decreases, coupled with increased lipolytic and catabolic activity due to insulin resistance that occurs at the time of pregnancy [11]. This results in decreased hydrolysis of triacylglycerols for storage in the mother and increase in placental lipolytic activity; available triacylglycerols are used by LPL to provide free fatty acids for foetal transport. Placental LPL hydrolyses triacylglycerols from posthepatic low-density lipoprotein (LDL) and very low-density lipoprotein (VLDL) but not the triacylglycerols that are present in chylomicrons [12].

In normal pregnancies, there are modifications in the lipid profile. A gestational increase in the concentration of triacylglycerols (TGs) of 300% and 25–50% for total cholesterol (TC) as well as variations in high-density lipoprotein (HDL) and low-density lipoprotein (LDL) levels have been described. This alteration in lipid concentration is a risk factor for developing PE. An increase in the risk of PE of up to 3.6 times has been reported when modifications in the lipid profile exist [13]. Physiologically, this gestational alteration is necessary to supply nutrients to the rapidly growing foetus and to meet maternal energy needs [14]. The increase in lipids and lipoproteins induces their accumulation in the arterial wall, forming atheroma plaques and thus mediating the genesis of atherosclerosis [15]. In cardiovascular diseases and PE under hypoxic conditions, the activation of enzymes that generate reactive oxygen species (ROS), such as NADPH oxidase and xanthine oxidase, is favoured, triggering an oxidative environment where oxygen and nitrogen free radicals develop. If these free radicals exceed the physiological defences, lipids can be oxidized. This oxidation is known as lipoperoxidation, and it can occur in three stages: mild, moderate or severe damage. When there is severe damage, highly toxic products, such as malondialdehyde (MDA), can be generated, which in turn damage low-density lipoprotein (LDL), generating the oxidized form (oxLDL). These oxidation processes damage both lipids and proteins. Protein damage can be due to protein carbonylation (mild damage), orthoquinone formation (moderate damage), and dityrosine formation (severe damage) [6].

### Lipoproteins

Plasma lipoproteins (LPs) are lipid-transporting particles that have an amphipathic structure, that is, both hydrophobic and hydrophilic moieties. LPs have an almost spherical morphology and contain a hydrophobic core formed by esterified cholesterol and triacylglycerols and a hydrophilic surface layer that contains unesterified cholesterol, phospholipids and proteins called apolipoproteins (Apos). Apos are involved in the binding of lipoproteins to their cell receptors, facilitating their uptake. Apolipoprotein A (Apo A) is the main polypeptide component of HDL and has two primary forms, namely, Apo AI, which constitutes 75%, and Apo AII, which represents the remaining 25%; in addition to Apo A, there are Apo B, C, D, E, F, G and M. The protein Apo M is a lipocalin found mainly in HDL responsible for hydrophobic binding molecules such as sphingosine-1-phosphate (S1P) [16]; its importance lies in maintaining HDL-induced vasorelaxation as well as promoting the vascular endothelial survival barrier. Chistoffersen et al. demonstrated in animal models that apoM is the physiological transporter protein for S1P in HDL and that apoM can deliver S1P to the S1P 1 receptor on endothelial cells. Thus, apoM-bound S1P mediates S1P1 receptor activation, resulting in vasoprotective effects [17]. The protein Apo B is the main component of LDL (80%) and accounts for 40% of VLDL (Table 3) [13, 16, 18, 19].

**Table 3:** Chemical compositions of lipoproteins

Chemical compositions of lipoproteins			
	% lipids	% Apo protein	Apo protein class
Chylomicrons	98	2	AI, AII, AIV, B, C, and E
VLDL	90	10	B-100, C, and E
IDL	80	20	B-100, C, and E
LDL	75	25	B-100
HDL	50	50	AI, AII, AIV, C, D, E and M

According to their chemical composition, size, and density, they are classified as high-density lipoproteins (HDL), low-density lipoproteins (LDL), intermediate-density lipoproteins (ILD), very-low-density lipoproteins (VLDL), and chylomicrons.

### Relationship between maternal and foetal lipoproteins

Maternal hyperlipidaemia during pregnancy favours the transport of lipids to the foetus [19]. However, increased or decreased transport of cholesterol to the foetus may have long-term consequences for the foetus [19]. The foetus has the ability to meet the demand for cholesterol from exogenous stores; the yolk sac early in pregnancy and later the placenta has the same property of storing cholesterol derived from the mother [20].

The human placenta requires cholesterol to synthesize progesterone; this hormone maintains the relationship between the foetus and the mother until successful completion of the pregnancy [21]. The placenta incorporates cholesterol mainly through the lipoproteins LDL and HDL obtained from the maternal bloodstream [19, 22]. To reach the foetal circulation, cholesterol must cross two barriers. First, cholesterol is acquired from the apical side of the syncytiotrophoblast (STS) as high-density lipoprotein (HDL), low-density lipoprotein (LDL) or very-low-density lipoprotein (VLDL). It is secreted along the basal side facing the villous stroma [23]. Subsequently, cholesterol is taken up by the endothelium of the foetal vasculature and transported to the foetal vessels. The proteins involved in the uptake of HDL, LDL, VLDL or unesterified cholesterol are scavenger class B receptor type 1 (SR-B1), cubulin, megalin, LDL receptor (LDLR) and Niemann-Pick-C1 (NPC1) proteins located on the apical or basal side of the STS or on the foetal endothelium. Through interaction with apolipoproteins (apoA1), cholesterol is released into the maternal or foetal circulation via ATP-binding cassette transporter (ABC) A1 and ABCG1 located on the apical/basal aspect of the placenta or in the endothelium [11, 24].

Maternal TG does not cross the placental barrier; instead, triacylglycerols in lipoproteins are hydrolysed by lipoprotein lipase present in the placenta. The resulting free fatty acids are transferred through the placenta by proteins that bind to fatty acids. Upregulation of this mechanism can increase the transport of fatty acids across the placenta to supply substrates to the foetal liver for the synthesis of TAG [25].

The placental transfer of fatty acids supports the growth and development of the foetus. Essential fatty acids, by their nature, cannot be synthesized in the body, and the foetus must obtain them from the mother through the placenta [26]. Long-chain polyunsaturated fatty acids (LC-PUFAs), such as arachidonic acid and docosahexaenoic acid (DHA), are generated from essential fatty acids. LC-PUFAs have specific functions in the composition of the plasmalemma and are particularly important for foetal neurodevelopment [27, 28]. Furthermore, fatty acids and their derivatives have essential signalling functions (eicosanoids) and act as receptor ligands. Given the importance of fatty acids, particularly LC-PUFAs, in the synthesis of membranes and as substrates for the generation of lipid mediators involved in signalling processes, it is likely that any deficiency in the maternal supply or the placental transfer will compromise foetal development [25].

The metabolism of lipoproteins in the foetus is still unknown. Likely, the circulating concentrations of maternal lipids available for placental uptake are not the primary determinant of the lipid profile of neonates. Changes in lipids in neonates may be an appropriate physiological response to an adverse intrauterine environment [14].

### Low-density lipoproteins (LDL)

LDL is of utmost importance because it guarantees a constant supply of cholesterol for cells and tissues. Cholesterol is necessary for synthesizing membranes, modulating membrane fluidity, and regulating cell signalling pathways. LDL is very heterogeneous in nature and varies in size, density and chemical composition. The chemical characteristics determine where LDL will be in the subendothelial space. LDL, having lipids as its main component, can undergo modifications by oxidation, enzymatic degradation, or lysis. These molecules have an apo-B100 protein region as a receptor ligand that binds to proteoglycans of the extracellular matrix through ionic interactions and becomes trapped in the subendothelium [7].

The oxidative modification of LDL is involved in the pathogenesis of different diseases. The acute atherosis observed in the spiral vessels in PE shares some resemblance to the atherosclerotic lesions of the coronary arteries [29]. The oxidation of LDL is a complex process during which both the protein and lipid fractions undergo oxidative changes that produce several complex products. Parthasarathy et al. described a great variety of products that are produced by the oxidation of LDL components [30]. Fatty acids can give rise to peroxides (13-hydroperoxylinolic acid), hydroperoxides (13-hydroxylininoleic acid), prostaglandin-like products (isoprostanes), aldehydes (malondialdehyde), and hydrocarbons.

Oxidation products can be formed from cholesterol and lysophosphatidylcholine, and other oxidative modifications of phospholipids can also occur. Protein oxidation originates from protein fragments, free carbonyls, modifications of various amino acid residues (cysteine, methionine, histidine, lysine, arginine, tryptophan, and tyrosine), and lipid-protein products or adducts that can be classified as lipofuscins. In turn, oxidative changes generate alterations in some particle properties, such as increased density, increased negative charge, and loss of enzymatic activities [31].

Oxidized LDL causes lesions in endothelial cells by positive regulation of the gene expression of adhesion molecules, facilitating the adhesion of monocytes and platelet aggregation and reducing the activity of endothelial nitric oxide synthase (eNOS), thus resulting in endothelial dysfunction [32]. High oxLDL concentrations can destabilize the messenger RNA (mRNA) of eNOS, causing a transcriptional reduction in eNOS levels [33].

oxLDL may also interfere with signal transduction from receptors associated with activation of eNOS (acetylcholine, bradykinin, serotonin, and histamine receptors).

Dysfunctional eNOS can generate superoxide anions that can react with NO, forming the peroxynitrite anion (ONOO-), a strongly nitrating and oxidizing reactive nitrogen species that promotes the oxidation of LDL [33].

The reduced availability of NO causes reduced vasodilation and consequently endothelial dysfunction, the main characteristic of preeclampsia, along with an increased cardiovascular risk [34].

When endothelial dysfunction occurs, the increased permeability of the vessel walls permits an increase in the penetration of LDL that exceeds the speed and efficiency of the reverse cholesterol transport system (TIC system mediated by HDL) associated with the process of returning it to the bloodstream. Collectively, these factors cause an increase in the time that lipoproteins remain within the subendothelial space, where they can undergo mild oxidation to produce minimally modified LDL molecules (MM-LDLs). Together with the oxidative stress present in the environment, the presence of angiotensin II and the reduction in flow pressure in areas prone to atherosclerosis, MM-LDLs are capable of activating nuclear factor kappa-β (NF-κβ), a transcription factor that increases the expression of molecules that participate in monocyte uptake steps (VCAM-1, ICAM-1, E-selectin, MCP-I, and IL-8). Once in the subendothelial space, monocytes transform into macrophages, which then oxidize MM-LDL to produce oxidized LDL. This process is favoured by angiotensin II and by the previous glucosidation of LDL. Macrophages take up oxidized LDL, a process mediated by macrophage colony-stimulating factor (MCSF) and stimulated by angiotensin II. Macrophages thus activated can in turn stimulate the cellular expression of angiotensin-converting enzyme (ACE) and the synthesis of angiotensin II, leading to a positive feedback loop. These macrophages will continue to take up lipids and experience an overload that causes degeneration until they become the so-called foam cells that will eventually die and release the lipids that will form the lipid nucleus, along with toxic substances, such as enzymes, free radicals, and superoxide anions. Toxic products injure the endothelium, which in some areas can even be destroyed and disappear. These processes increase endothelial dysfunction [35].

In addition, oxLDL stimulates the production of antibodies (AB-oxLDL). Increased circulating LDL levels have been shown to be a marker of oxidative stress in PE. Although the role of Ab-oxLDL is not clear, some research groups have suggested that an increase in these antibodies is indirect evidence of oxidative stress, as they have shown that oxLDL and Ab-oxLDL are higher in PE than in normal pregnancy [36].

High concentrations of LDL in women with PE are related to low birth weight; this has been associated with a persistent decrease in the activity of LDL receptors and subsequent alterations in foetal liver growth. PE can lead to undernourishment of the foetus secondary to uteroplacental vascular insufficiency. The foetus responds to this state (during the third trimester), maintaining brain growth at the expense of body growth and affecting the liver, which overgrows in the last trimester, affecting the metabolism of LDL.

### High-density lipoproteins (HDL)

HDL comprises triacylglycerols, esterified and unesterified cholesterol, phospholipids, and 50% proteins, and its concentration must remain high (> 57 mg/dL) because it exerts a beneficial actions at the level of the arteries, counteracting the harmful actions of LDL and eliminating cholesterol from the arterial layers in the form of bile acids [37]. HDL is considered a predictor of cardiovascular risk [38, 39]. Huang et al. showed a U-shaped relationship between HDL concentrations and mortality; when HDL concentrations < 63 mg/dL are associated with an increased risk of mortality from any cause, concentrations < 46 mg/dL are associated with cardiovascular death, and < 70 mg/dL is associated with cancer mortality. However, concentrations > 80 mg/dL HDL are also associated with mortality from any or a specific disease [40].

HDL is produced in the liver (approximately 70%) and intestine (approximately 30%). HDL is formed as nascent particles with intestinal and hepatic secretion of apolipoprotein AI (apo AI) or by their release from lipoproteins rich in triacylglycerols. Subsequently, apo A-I captures phospholipids from cells forming discoidal HDL particles known as pre β-HDL [41]. These HDL particles interact with the AB-CA1 (ATP-binding cassette transporter class A, type 1) transporter on the surface of cells, capturing more phospholipids and unesterified cholesterol. Next, the enzyme LCAT (lecithin-cholesterol acyltransferase) esterifies cholesterol and mobilizes it towards the centre of the HDL particles, which acquire a larger size and spherical shape and are then called a-HDL. HDLs complete their maturation by capturing more unesterified cholesterol of cellular origin through the action of the HDL receptor SR-BI (scavenger receptor class B, type I) and the transporter ABCG1 (ATP-binding cassette subfamily G member 1). Mature HDLs are classified into HDL2 and HDL3, which are of higher density, smaller size, and lower cholesterol content [42, 43]. Mature HDL is known as HDL-2 (density 1.063–1.12 g/mL) and is classified into subvarieties (HDL-2a and 2b). The newly formed HDLs are known as HDL-3 (density 1.12–1.21 g/mL), which in turn are classified as HDL-3a, 3b and 3c. They can also be classified by the type of apolipoproteins they contain. Sixty percent of them contain ApoA-I and ApoA-II proteins (two molecules of each apoprotein for each HDL). The rest contain only apoA-I protein (four molecules of apoA-I per HDL). HDL containing ApoA-I protein and ApoA-II protein are present in HDL-3; those containing ApoA-I protein are found in HDL-2 and 3 [43, 44].

The PON-I enzyme present in the HDL particles provides it with important protective activity. PON is expressed in different tissues: the PON-3 and PON-I genes are expressed in the liver, while PON-2 is expressed in the brain, liver, kidney, and testis. PON-I and PON-3 are secreted by liver cells, and in circulation, they are bound to HDL. The PON-2 enzyme is exclusively cellular in location. It is found in the membrane with its active site towards the outside of the cell. PON-I also exhibits the same orientation on the cell membrane before being excreted into the serum. PON-2 and PON-3 exhibit antioxidant properties and protect or reverse the oxidation of HDL and LDL. However, under oxidative stress conditions, HDL function can be impaired, leading to inactivation of the PON-I enzyme [45].

The loss of this enzyme decreases the protective and anti-inflammatory capacity of HDL. HDL has been described as a particle with powerful atheroprotective properties; it reduces coronary atherosclerosis by reducing the expression of adhesion molecules in endothelial cells and therefore reduces inflammation. HDL is involved in improving the endothelial synthesis of NO and improving endothelial function [46]. However, in patients with cardiovascular diseases, HDL becomes dysfunctional and not only is characterized by the loss or reduction of its normal function but can also become proatherogenic; HDL does not inhibit the oxidation of either LDL or itself, it is unable to prevent the chemotactic effect of LDL in monocytes, it cannot stimulate nitric oxide production, and it loses the ability to limit macrophage adhesion to the vascular wall. On the other hand, HDL has the function to join with lectin-type receptors of oxidized LDL (LOX-I) and Toll-like receptors [47].

The consequences of dysfunctional HDL include loss of normal biological functions, induction of the proinflammatory state, macrophage adhesion to the vascular wall, an increase in atherosclerotic lesions, and other complications that cause endothelial dysfunction [7].

In the foetus, the metabolism of HDL is different from that in adults. HDL forms a complex with apolipoprotein E and is taken up by cells through the lipoprotein receptor (LRP) [19]. High maternal concentrations of HDL may be involved in the transfer of cholesterol from the placenta to the foetal circulation mediated by the AB-CA1 transporter, which is highly expressed in this tissue. HDL is rich in apolipoprotein E, which is essential for redistributing cholesterol from the tissues where it is present in high concentrations to the tissues that need cholesterol for their metabolic processes. The increase in maternal lipid intake in PE may produce changes in the levels of lipids that cross the placenta, leading to changes in neonatal lipid and lipoprotein concentrations. The human placenta expresses lipoprotein receptors in large quantities, and HDL receptors play a relevant role in the uptake of maternal lipoproteins for placental steroid metabolism. During the first trimester, trophoblastic cells express SR-BI (an HDL receptor), which may serve as an effective route to deliver maternal lipoprotein cholesterol esters to foetal tissue [14].

### Lipoproteins and foetal programming: Developmental Origins of Health and Disease (DOHaD)

The adverse intrauterine environment could serve as a stimulus for altering postnatal health status and lead to greater susceptibility to developing long-term diseases. This is currently known as the Developmental Origins of Health and Disease (DOHaD) concept. Several clinical, epidemiological, and basic science studies have measured the magnitude of early foetal programming and later disease risk [48].

Metabolic changes in intrauterine development can establish long-term physiological and structural patterns, which program health during adult life; however, diseases such as atherosclerosis, arterial hypertension, cerebrovascular accidents, type 2 diabetes mellitus, and dyslipidemia can also be programmed [49].

The placenta is an organ that regulates the passage of nutrients and products of metabolism, including oxygen, from the maternal circulation to the foetus and vice versa. This transfer will depend directly on the mother’s nutritional status, the foetus’s genetic load and the growing foetus’s requirements [50].

In pregnancy, when there is inadequate remodelling of the spiral arteries, a hypoxic environment is generated that triggers a complex cascade of events that affect the normal development and growth of the foetus. Hypoxia activates multiple responses regulated by hypoxia-inducible factor I (HIF-I), which is primarily involved in the cellular control of oxygen consumption and release, inhibition of growth and development, and promotion of anaerobic metabolism. Hypoxia is an important physiological process for the development of the foetus and is involved in different processes during embryogenesis, including placentation, angiogenesis, haematopoiesis, and foetal growth trajectory. It modulates the expression of genes by epigenetic mechanisms and determines the health status in adult life [8]. Interestingly, oxidative damage to LDL and HDL has been associated directly or indirectly with foetal programming.

León-Reyes et al. found an increase in biomarkers of lipid damage concentrated in lipoproteins of neonates from mothers with PE [7]. Conjugated dienes (DCs), a biomarker for mild damage, were present in 23.3% of PE pregnancies and 19.9% of normal pregnancies. Lipohydroperoxides and moderate damage markers were 82.4% in PE and 21.1% in normal pregnancies, and for severe damage, the determination of malondialdehyde (MDA) was 103.8% and 51.5%, respectively. Likewise, those researchers evaluated the damage to proteins and observed a significant difference only in protein carbonylation. They suggested that the protection of proteins could be explained by the total antioxidant capacity, which is 40% greater in the neonates of mothers with PE than in neonates of healthy mothers.

On the other hand, PON-I activity in umbilical cord plasma has been reported to be lower in neonates of women with PE than in neonates of healthy women (*p* < 0.0015) [7].

Kahramaner et al. reported a homocysteine level of 8.2 ± 5.9 µmol/L in children of mothers with PE, unlike in the plasma of children of healthy mothers (5.3 ± 2.7 µmol/L). Homocysteine acts on the walls of blood vessels, and high concentrations can cause endothelial dysfunction and are associated with a wide variety of vascular disorders, such as cerebrovascular events and atherosclerosis [51].

Additionally, a higher concentration of endothelin (ET), a potent vasoconstrictor agent released in the vascular endothelium that has been found to contribute to myocardial injury in some neonates, has been reported in the umbilical cord of children from women with PE [52].

The presence of the HDL APOE-e4 allele in preeclamptic mothers was associated with an approximately eightfold higher risk of preeclampsia (adjusted OR = 8.4; 95% CI: 2.51 to 28.17; *p* = 0.001). In addition, higher concentrations of triacylglycerides and LDL and lower HDL concentrations were found in pregnant women with severe preeclampsia whose newborns were carriers of the APOE-e4 allele. Similarly, higher lipid concentrations are seen in newborns, which could be associated with a risk of dyslipidamia in adulthood [53]. In this context, newborns of mothers who have PE, due to dysregulation in the production of or damage to key molecules required for a healthy foetal environment, are at risk of neurological diseases; disabilities such as cerebral palsy, retardation, sensory deficiencies or behaviour disorders; metabolic diseases such as diabetes, hypertension, metabolic syndrome, and dyslipidaemia; and cardiovascular diseases such as coronary heart disease, cerebrovascular disease, and peripheral vascular disease [54, 55].

## Discussion

Preeclampsia continues to be one of the main complications of pregnancy and a cause of maternal death worldwide. Despite the great number of research efforts focused on studying its aetiology, its aetiology remains unclear. In PE, placentation is ineffective, and a vascular medium of low capacitance and high resistance is created, generating a state of local hypoxia. This state activates several enzymes, such as xanthine oxidase and nicotinamide adenine dinucleotide phosphate (NADPH) oxidase, resulting in increases in the concentration of the superoxide anion (O_2_•-), which can form hydroxyl radicals (HO•) that cause damage to biomolecules such as lipids and proteins [7].

HDL and LDL can undergo oxidative damage, generating highly toxic products within cells and molecules circulating in the intravascular space.

If lipoproteins are affected, will they reach the foetal circulation? What antioxidant mechanisms does the newborn have to combat the damage? These are some of the questions we tried to address in this review.

It has been shown that PON-I activity (in essence, an antioxidant) is lower in neonates of women with PE than in neonates of healthy women.

The levels of other molecules, such as homocysteine, endothelin and lipids, in the neonates of women with PE are higher than those in children of healthy mothers. This environment can condition neonates to develop endothelial dysfunction or even atherosclerosis [51].

When preeclampsia manifests before 34 weeks of gestation, it causes the neonate to have low birth weight. However, if PE ma nifests after 37 weeks of gestation, foetal complications are reduced [56]. Therefore, the environment in which the foetus develops can be the origin of health or disease and can condition the children born to women with PE to develop dyslipidemia as well as cardiovascular, neurological and chronic degenerative diseases in their adult lives.

### Strengths and limitations of the review

This review addresses current knowledge about the impact that preeclampsia generates on HDL and LDL lipoproteins in neonates born to women with this syndrome. The analysis of these molecules and their subpopulations, especially those of HDL, showed that they present modifications due to oxidation processes. These modifications include “dysfunctional HDL”, a situation that may be closely linked to the pathophysiology of preeclampsia. In addition to the impact that this generates on the foetus, it can possibly contribute to the development of diseases throughout the life of the neonate due to foetal programming. This term that still requires long-term follow-up studies. However, there are few studies aimed at monitoring newborns showing that the children of mothers with preeclampsia develop diseases throughout their lives, which prevents conclusive statements about the real impact of preeclampsia on the newborn.

## Conclusion

In clinical syndromes such as PE, biomolecules such as lipoproteins can suffer damage or oxidative modifications that will be transmitted to the foetus. However, the total antioxidant capacity in the neonates of women with PE is favoured. Lipoproteins and their products are involved in key signalling processes; therefore, any deficiency in the maternal supply or in the placental transfer of these molecules to the foetus will compromise its development. Many studies have agreed that the origin of the development of health or disease may depend on the intrauterine environment in which the foetus develops. If the environment is unfavorable, it could program the neonate in their adult life to develop metabolic, neurological and cardiovascular diseases. The clinical relevance of this review lies in having consistent data on the damage received by various molecules, such as LDL and HDL lipoproteins, during pregnancy with PE to broaden the knowledge of this syndrome and have tools that allow us to implement therapeutic strategies to reduce the risk of maternal–foetal death and in turn improve foetal development and the quality of life of the newborn. A clear example could be the development of therapies through the use of HDL biomarkers for their beneficial functions, as they are responsible for transporting multiple proteins and lipids with bioactive properties that favourably influence various biological processes. Much remains to be discovered; thus, future perspectives suggest developing prospective studies in which neonates who underwent embryonic development with PE are strictly followed up to confirm whether oxidative modifications in lipoproteins persist and whether they significantly affect the quality of life in adulthood.

## Data Availability

Not applicable.

## Abbreviations

PE: Preeclampsia
LDL: Low-density lipoprotein
HDL: High-density lipoprotein
oxLDL: Oxidized low-density lipoprotein
eNOS: Endothelial nitric oxide synthase
TG: Triacylglycerides
TC: Total cholesterol
ROS: Reactive oxygen species
MDA: Malondialdehyde
LP: Plasma lipoprotein
APO: Apolipoprotein
IDL: Intermediate-density lipoprotein
VLDL: Very-low-density lipoprotein
STS: Syncytiotrophoblast
SR-B1: Scavenger receptor class B type 1
NO: Nitric oxide
DOHaD: Developmental Origins of Health and Disease
